# An artificial intelligence approach to predicting personality types in dogs

**DOI:** 10.1038/s41598-024-52920-9

**Published:** 2024-01-29

**Authors:** Mohammad Hossein Amirhosseini, Vinaykumar Yadav, James A. Serpell, Piya Pettigrew, Paris Kain

**Affiliations:** 1 Department of Computer Science and Digital Technologies, School of Architecture, Computing and Engineering, University of East London, London, UK; 2 School of Veterinary Medicine, University of Pennsylvania, Philadelphia, PA USA; 3 Dogvatar, Inc., Miami, FL USA

**Keywords:** Animal behaviour, Computational science

## Abstract

Canine personality and behavioural characteristics have a significant influence on relationships between domestic dogs and humans as well as determining the suitability of dogs for specific working roles. As a result, many researchers have attempted to develop reliable personality assessment tools for dogs. Most previous work has analysed dogs’ behavioural patterns collected via questionnaires using traditional statistical analytic approaches. Artificial Intelligence has been widely and successfully used for predicting human personality types. However, similar approaches have not been applied to data on canine personality. In this research, machine learning techniques were applied to the classification of canine personality types using behavioural data derived from the C-BARQ project. As the dataset was not labelled, in the first step, an unsupervised learning approach was adopted and K-Means algorithm was used to perform clustering and labelling of the data. Five distinct categories of dogs emerged from the K-Means clustering analysis of behavioural data, corresponding to five different personality types. Feature importance analysis was then conducted to identify the relative importance of each behavioural variable’s contribution to each cluster and descriptive labels were generated for each of the personality traits based on these associations. The five personality types identified in this paper were labelled: “Excitable/Hyperattached”, “Anxious/Fearful”, “Aloof/Predatory”, “Reactive/Assertive”, and “Calm/Agreeable”. Four machine learning models including Support Vector Machine (SVM), K-Nearest Neighbour (KNN), Naïve Bayes, and Decision Tree were implemented to predict the personality traits of dogs based on the labelled data. The performance of the models was evaluated using fivefold cross validation method and the results demonstrated that the Decision Tree model provided the best performance with a substantial accuracy of 99%. The novel AI-based methodology in this research may be useful in the future to enhance the selection and training of dogs for specific working and non-working roles.

## Introduction

In the field of psychology, the term ‘personality’ generally refers to relatively consistent patterns of thinking, feeling, and behaving that make up an individual’s unique character and which are shaped by both genetic and environmental factors^1^. ‘Temperament’ is a related but distinct concept that is often used interchangeably with personality^2–4^, as it is in the current paper.

Canine personality/temperament plays a critical role in establishing and maintaining positive, functional relationships between humans and domestic dogs (*Canis familiaris*). Dogs who display undesirable temperament traits are at greatly increased risk of being euthanized during their lifetimes^5^. Nearly 50% of people surrendering dogs to animal shelters in the USA cite behavioral problems as a contributory factor and roughly a quarter cite them as the primary reason for relinquishment^6–10^. In addition, many dogs suffer from chronic fears and anxiety states that may not necessarily result in relinquishment or euthanasia, but which undoubtedly impair the overall welfare of these animals^11^. Important public health concerns also arise from canine personality traits.

According to the Centers for Disease Control and Prevention and the Humane Society of the United States, there are about 4.7 million dog bites every year in the U.S. and these bites result in approximately 16 fatalities^12^.

In the area of specialized working dogs, behavioral and personality characteristics are key factors determining the suitability of individual dogs for specific working roles. More than any other domestic species, the dog’s extraordinary diversity of breeds and types reflects a long history of selection for behavioral traits and attributes that have adapted these animals to the performance of specific useful activities or tasks ranging from hunting, guarding, and detection work to the provision of companionship and social support^13^.

For all the above reasons, many researchers have attempted to develop reliable and valid personality or temperament assessment tools for domestic dogs^2,3,14^. Some of these assessment methods aim to quantify the behavior of dogs directly, either by recording their responses in standardized test batteries or by observing spontaneous expressions of behavior in various relevant contexts^3,15,16^. Others seek to evaluate canine personality or temperament by proxy by inviting dog owners, trainers, and handlers to complete questionnaires describing dogs, either in terms of appropriate adjectives (e.g., excitable, playful, assertive, etc.)^17,18^, or by indicating the animals’ typical responses to common stimuli and scenarios using a series of Likert-type rating scales^2,19–21^. The latter approach has the advantage that it allows the assessment of very large numbers of dogs for minimal cost and effort and is more likely to record relatively uncommon behavioral responses that would likely be missed in single tests or observation periods or using simple personality descriptors. Such methods sometimes attract criticism for being too “subjective”, although subjective biases can be reduced by asking respondents to refer to specific behavior in well-defined eliciting contexts and situations. Also, when large samples are available and aggregated, the effects of individual response biases are greatly reduced^2^.

Probably the most widely used proxy measure of canine temperament is the Canine Behavioral Assessment & Research Questionnaire (C-BARQ) developed at the University of Pennsylvania^19,22^. The questionnaire comprises 100, 5-point, ordinal rating scales addressing the frequency or severity of dogs’ behavioral responses to a wide range of common situations and stimuli that most dogs are likely to encounter during their daily lives. The C-BARQ has been in circulation as a research tool for 20 years and has helped to generate a substantial list of scientific publications (see: https://vetapps.vet.upenn.edu/cbarq/published-articles.cfm). The instrument’s various scales have been shown to have adequate internal reliability and acceptable test-retest and inter-rater reliability^22–24^. Construct and criterion validity of the C-BARQ have been established by demonstrating associations with: (a) clinical diagnoses of behavior problems in companion dogs^19^, (b) training outcomes in working dogs^15,22,25^, (c) the behavior of dogs in standardized test batteries^16,26–31^, (d) neurophysiological markers of canine anxiety disorders^32,33^, and (e) genetic loci known to be associated with the brain and behavior^34–36^.

While the C-BARQ was designed originally to investigate the prevalence and severity of behavior problems in dogs, rather than as measure of personality *per se*^19^, the breadth of its behavioral coverage and the similarity of many of its questionnaire items to those of other canine personality assessments^14^ suggests that it provides a suitable method to evaluate personality in dogs. Furthermore, a recent study was successful in using C-BARQ data to generate underlying personality subtypes or groupings of dogs using Latent Class Analysis^37^.

A wide variety of human personality assessment tools is currently available. The Myers Briggs Type Indicator (MBTI) and Big Five Inventory (BFI) are the two most commonly used and validated tools for studying individual personality traits in humans and for grouping people into categories based on consistent, individual styles of behaving, thinking, and feeling^38,39^. Much of the research in this area has focused on measuring personality attributes to provide career exploration and vocational guidance that fits with these personality attributes^40,41^.

More recently, artificial intelligence (AI) and machine learning (ML) techniques have been widely and successfully used for classifying and predicting human personality types based on these different personality profiling tools^38^. Researchers in different fields such as Social Science and Natural Language processing have shown significant growing interest in automated personality prediction using textual data and social media^42^. In fact, the application of conventional personality analyses has mostly been limited to clinical psychology, counselling, and human resource management. However, automated personality type prediction from textual data and social media has extensive applications, including but not limited to social media marketing or dating applications and websites^43^.

Golbeck et al.^44^ conducted one of the earliest studies on personality prediction using machine learning techniques. By analyzing the contents of people’s ‘tweets’ they were able to predict their personality types accurately based on MBTI. In another study, the Naïve Bayes and Support Vector Machine (SVM) techniques were used to predict an individual’s personality type based on their word choice^45^. The database used in this study was built from writing samples taken from 40 graduate students along with their MBTI personality type. The performance of these two techniques was compared and the results showed that the Naïve Bayes technique performs better than SVM on this small dataset. Two years later, Wan et al.^46^ successfully predicted Big Five personality types of Weibo (a Chinese social network) users by analyzing their texts using a machine learning model. Tandera et al.^47^ used a deep learning architecture to predict the Big Five personality types of individuals based on the information on their Facebook pages. They proved that the performance of their deep learning model successfully outperformed the accuracy of previous similar studies that used traditional machine learning models. In another study, various types of recurrent neural networks (RNNs) such as simple RNN, gated recurrent unit (GRU), long short-term memory (LSTM) and Bidirectional LSTM were used to build a classifier capable of predicting the MBTI personality type of an individual based on their social media posts^48^. Their results showed that among these models, LSTM gave the best results. Furthermore, Cui and Qi^49^ used three machine learning models including Logistic Regression, Naïve Bayes, and SVM to predict the MBTI personality type of an individual based on their social media posts. Their results showed that SVM performed better than the other two models. More recently, Amirhosseini and Kazemian^38^ implemented an Extreme Gradient Boosting model for personality type prediction based on the MBTI. Their results showed that the performance of their model outperformed all other existing models that were using the same dataset. The dataset used in this study was the publicly available Myers–Briggs Personality Type dataset on Kaggle containing 8675 rows of data and two variables. The first variable is for the MBTI personality type of a given person, and the second variable includes fifty posts obtained from the individual’s social media which have been separated by three pipe characters^50^. So far, this has been the most successful model for predicting the MBTI personality type of a person.

Similar approaches have not yet been applied to data on canine personality/temperament although, given the wide variety of working and non-working “careers” occupied by dogs in modern society, this would appear to be a potentially productive new area of research.

The present paper describes an initial attempt to apply AI and ML techniques to the classification and prediction of canine personalities using behavioral data derived from the C-BARQ database at the University of Pennsylvania. We also consider the extent to which the resulting personality types make sense from a biological perspective and discuss the possible applications of this methodological approach to the future selection and training of dogs for specific roles.

## Methodology

### Source of data

The data used in this research were derived from the C-BARQ database at the University of Pennsylvania School of Veterinary Medicine (https://vetapps.vet.upenn.edu/cbarq/). The C-BARQ (Canine Behavioural Assessment & Research Questionnaire) is an online survey instrument designed to allow dog owners, handlers, and professionals to provide standardized evaluations of canine temperament and behaviour^19,22^. The reliability and validity of these behavioural assessments have been confirmed in multiple studies (see https://vetapps.vet.upenn.edu/cbarq/published-articles.cfm for a recent list of published studies). At the time writing, the C-BARQ database contains behavioural records for 70,122 dogs that are freely available for collaborative research. The behavioral items in the C-BARQ comprise 100 questions addressing dogs’ responses to a wide variety of common situations and stimuli (see^22^).

The C-BARQ dataset is not a labelled dataset as there is no target variable. Consequently, an unsupervised machine learning algorithm was used to perform clustering using only input vectors without referring to known or labelled outcomes. Each cluster will refer to a collection of data points (dogs) aggregated together because of certain similarities.

### Pre-processing and data cleaning

Data cleaning was performed prior to implementing the machine learning models to avoid significant errors and inappropriate clustering. All samples with missing values for one or more attributes were removed from the dataset. When the data cleaning process was completed, there were 7807 complete samples remaining in the dataset.

### Data encoding

Out of 157 remaining attributes, 133 were identified as numerical attributes as they were including values with integer type. The other 24 attributes were identified as non-numerical attributes as they were including values with string type. As a result, data encoding had to be conducted in order to convert these values to numerical values which can be used for training the machine learning models. ‘LabelEncoder’ function from Scikit-Learn library in Python was used to perform encoding process.

### Feature selection for clustering

As the main goal in the current research was creating a set of personality traits for dogs based on their behavioural patterns, and to develop an AI-powered personality prediction tool for dogs, only the 100 scored behavioural items in the C-BARQ dataset were selected for clustering.

### Clustering approach

As the dataset was not a labelled dataset, a clustering approach was used. K-Means algorithm was used in this research which is an unsupervised learning algorithm. This algorithm groups the unlabelled dataset into different clusters. It starts with a first group of randomly selected centroids, which are used as the beginning points for every cluster, and then performs iterative calculations to optimize the positions of the centroids^51^. The main goal is to define k centroids, one for each cluster. Placing these centroids can be difficult because different locations create different results. Thus, they should be placed as far away from each other as possible. In the next step, the algorithm takes each data point and associates it to the nearest centroid. When no point is pending, this step is completed and an early group is identified. Following this step, it is necessary to re-calculate k new centroids as centers of the clusters resulting from the previous step. After deciding about these k new centroids, a new data point association needs to be done between the same data points and the nearest new centroid. This process will be repeated in a loop and the algorithm may notice that the k centroids change their location in each iteration until no more changes occur. In other words, the centroids do not move any further. Finally, the algorithm aims at minimizing an objective function, in this case a squared error function. Suppose there is a set of observations $({x}_{1}, {x}_{2}, {x}_{3}, \dots , {x}_{m})$. As a result, the objective function will be:

1 $$W\left(S, C\right)= \sum_{i=1}^{m}\sum_{k=1}^{K}{\omega }_{ik}{\Vert {x}_{i}-{c}_{k}\Vert }^{2}$$

2 $$where\left\{\begin{array}{l}{\omega }_{ik}=1 \;if\; {x}_{i} \;is \;in \;cluster\; k\\ {\omega }_{ik}=0 \;otherwise\end{array}\right.$$

3 $$c_{k} \;is\; the\; centroid\; of\; x_{i} \;s\; cluster.$$

In fact, the algorithm is trying to mathematically solve a minimization problem that consists of two parts. First, the algorithm minimizes *C with respect to* ${\omega }_{ik}$ *with* ${C}_{k}$ fixed. Second, it minimizes *C with respect to* ${C}_{k}$ *with* ${\omega }_{ik}$ fixed. This is shown in the below equations:

First step:

4 $$\frac{\partial C}{\partial {\omega }_{ik}}={\sum }_{i=1}^{m}{{\sum }_{k=1}^{K}\Vert {x}_{i}-{c}_{k}\Vert }^{2}$$

5 $$where,{\omega }_{ik}=\left\{\begin{array}{c}\begin{array}{cc}1,& ifk={\mathit{argmin}\Vert {x}_{i}-{c}_{j}\Vert }^{2}\end{array}\\ \begin{array}{cc}0,& otherwise\end{array}\end{array}\right.$$

Second step:

6 $$\frac{\partial C}{\partial {c}_{k}}=2\sum m{\omega }_{ik}\left({x}_{i}-{c}_{k}\right)=0$$

7 $$where,{c}_{k}=\frac{{\sum }_{i=1}^{m}{\omega }_{ik}{x}_{i}}{{\sum }_{i=1}^{m}{\omega }_{ik}}$$

To reach the point where the centroids no longer change, the algorithm must pay attention to the choice of K value. However, determining the initial value of K is challenging. To address this challenge, the performance of the algorithm should be calculated for different numbers of centroids. The distance between the data point and the centroid of each cluster can be calculated as long as convergence occurs. Then all the calculated distances should be added up as a performance indicator. The size of the objective function will decrease when the number of cluster centroids increases. The Elbow method can be used to select the best K value in this algorithm.

### Elbow method

**Figure 1:**
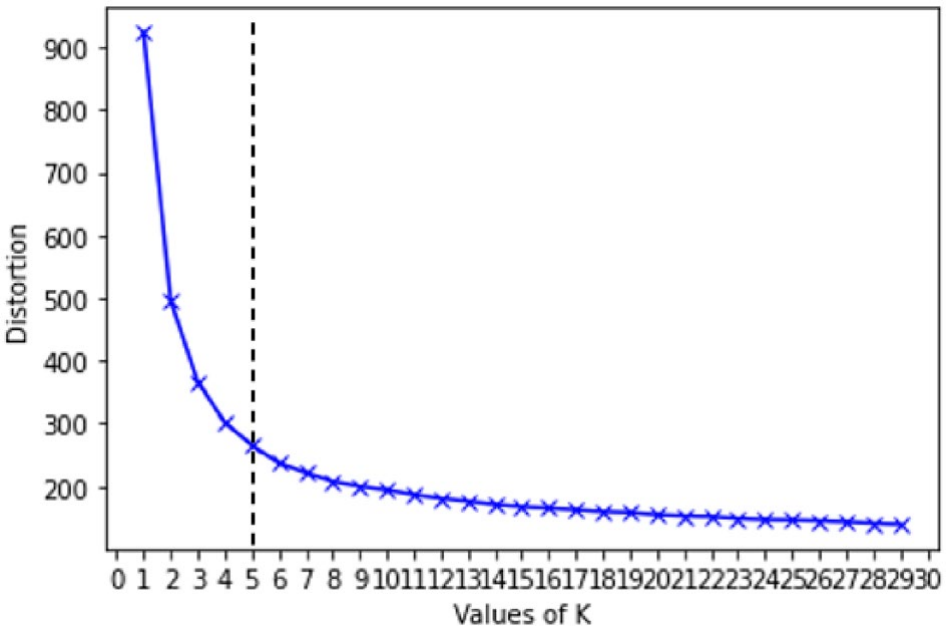
Demonstrating the Elbow diagram.

The Elbow method is a visual approach to selecting the optimal number of clusters by fitting the model with a range of values for K. A line chart will be created that resembles an arm and the ‘elbow’ which is the point of inflection on the curve, would be a good indication that the underlying model fits best at that point. In this research, the *KElbowVisualizer* from *yellowbrick* Python library was used to fit the K-Means model for a range of K values from 2 to 30 on the dataset. The process starts with K = 2 and keeps increasing it by 1 in each step. The scoring parameter *metric* was set to distortion, which computes the sum of squared distances from each point to its assigned centre. The average distance drops dramatically, and after that it reaches a plateau when K value increases further. Figure 1 demonstrates that, when the model is fit with 5 clusters, a line annotating the ‘elbow’ can be seen in the graph, which is the optimal number of clusters. In other words, there is a sharp fall of average distance when k is in the range of 1–5. After k = 5 the slope is relatively smooth. As a result, 5 was chosen as the best value of k.

### Machine learning classifiers to predict the dog’s personality type

Four different machine learning models were implemented to predict the personality traits of dogs. The models included Support Vector Machine (SVM), K-Nearest Neighbour (KNN), Naïve Bayes, and Decision Tree.

The train_test_split() function from the sklearn library was used to split the new labelled dataset into training and testing sets. 70% of the data was used for training and 30% was used for testing the models. The hyperparameter tuning was performed to optimise the performance of implemented machine learning classifiers. Table 1 shows the parameters for each classifier and the values set for each parameter.

**Table 1:** Hyperparameter tuning for each classifier.

Classifier	Parameters	Value
SVM	*C*	*1*
	*kernel*	*rbf*
	*degree*	*3*
	*gamma*	*scale*
	*shrinking*	*TRUE*
	*tol*	*0.001*
	*cache_size*	*200*
	*max_iter*	*1*
	*decision_function_shape*	*ovr*
	*random_state*	*42*
KNN	*n_neighbors*	*5*
	*weights*	*uniform*
	*algorithm*	*auto*
	*leaf_size*	*30*
	*p*	*2*
	metric	*minkowski*
Decision Tree	*criterion*	*‘gini’*
	*splitter*	*‘best’*
	*min_samples_split*	*2*
	*min_samples_leaf*	*1*
Naïve Bayes	*var_smoothing*	*1.00E−09*
	*priors*	*None*

The models were evaluated using a five-fold cross validation method and their performance was compared to find the most efficient classifier for prediction of dog’s personality type.

### Feature importance analysis

Feature importance analysis was conducted to identify the relative importance of each C-BARQ behavioural variable’s contribution to each cluster (see Tables 3, 4, 5, 6 and 7). The cut-off was set arbitrarily to the top 20 most important behavioural attributes defining each cluster. To provide an appropriate descriptive label for each of these personality types, the 20 most influential C-BARQ variables derived from the feature importance analysis were used. The threshold was set at 20 because the feature importance diagrams (See Figures 3, 4, 5, 6, 7) tend to have a more gradual slope after the first 20 most important features while the remaining features do not contribute substantially to the model.

To determine the direction of behavioural effects in each cluster, mean values were calculated for the 20 most influential C-BARQ variables in each cluster, and the results compared with the mean values of the same variables in the other 4 clusters combined. Based on these comparisons, appropriate behavioral descriptors could be applied to each personality grouping. These descriptors and the calculated mean values are also presented in Tables 3, 4, 5, 6 and 7.

## Results

### Clustering model to identify the dog’s personality type

**Figure 2:**
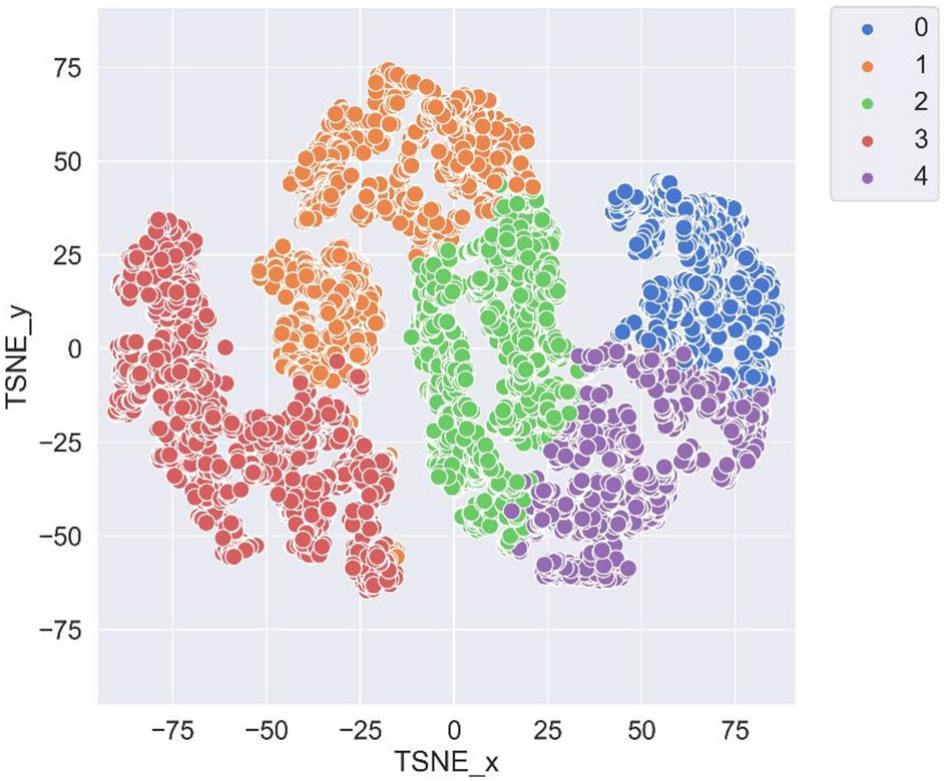
TSNE plot of samples in different clusters.

Figure 2 shows a TSNE (t-distributed stochastic neighbour embedding) plot created after performing K-Means clustering. TSNE plot is a statistical method for visualising high-dimensional data by giving each datapoint a location in a two-dimensional map. This figure demonstrates the distribution of samples (dogs) and how they are separated from each other into different clusters. The clusters are labelled by number from 0 to 4. Accordingly, a new column was added to the dataset as the target variable containing the relevant cluster number (label) for each sample. Table 2 shows the number of samples in each cluster.

**Table 2:** Number of samples in each cluster.

Cluster ID	Number of samples
0	1676
1	934
2	1626
3	864
4	2707

**Figure 3:**
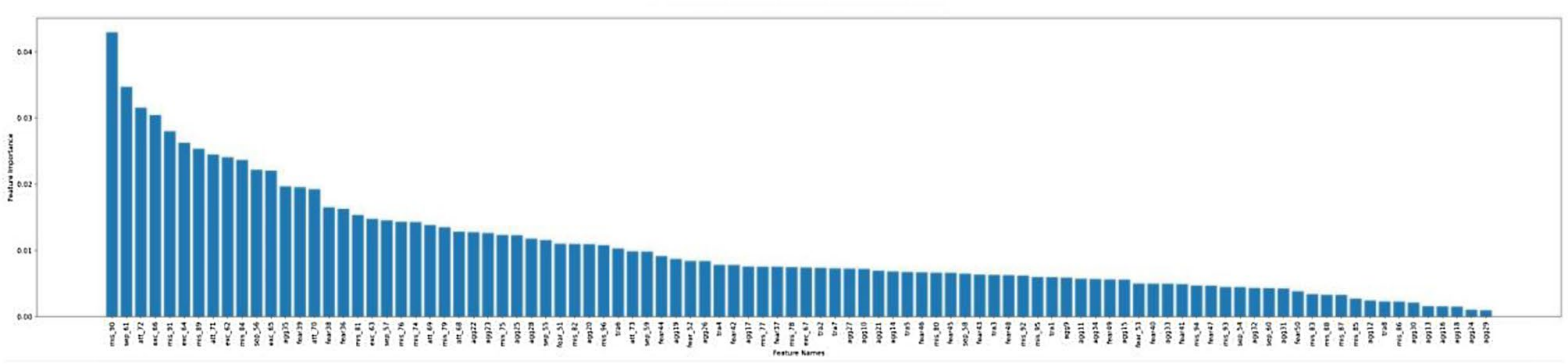
Feature importance for cluster 0.

**Figure 4:**
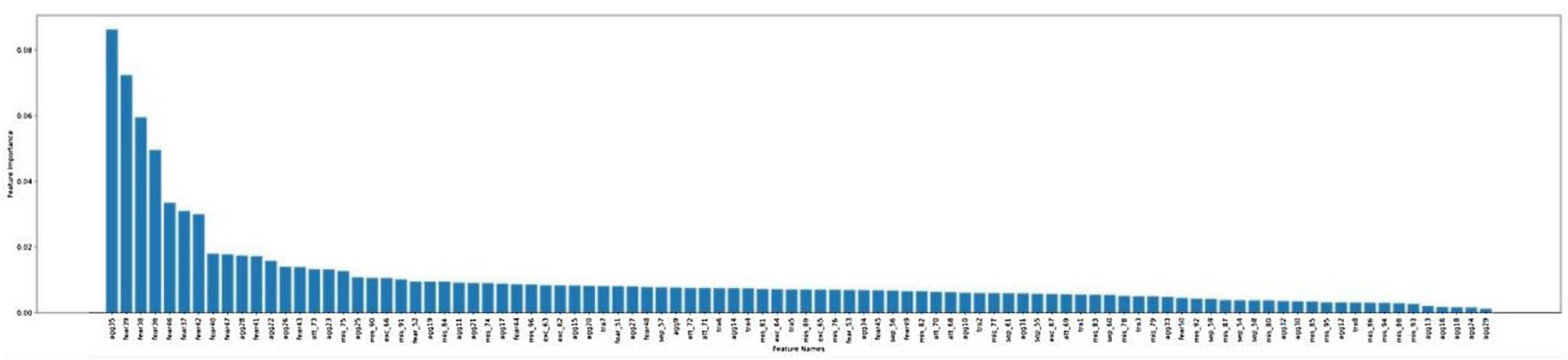
Feature importance for cluster 1.

**Figure 5:**
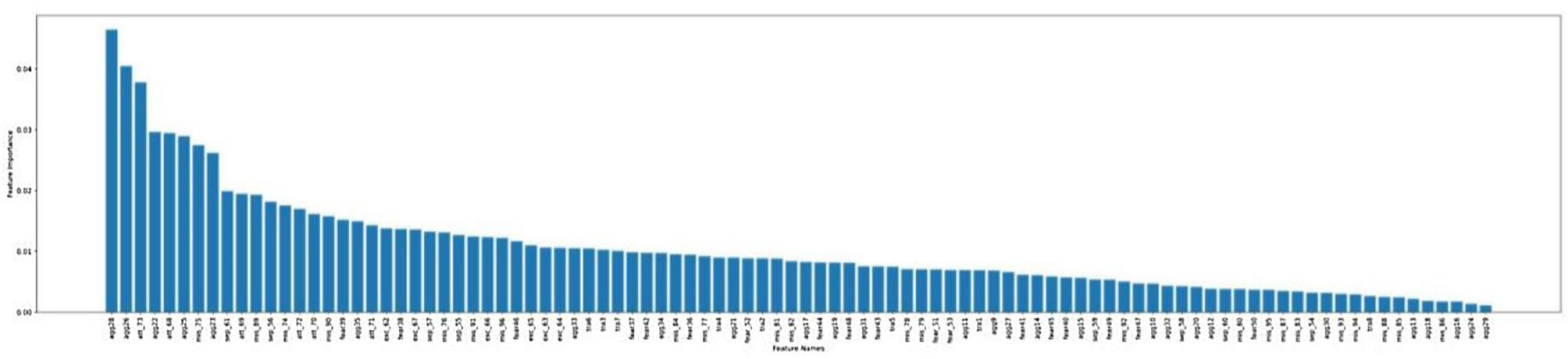
Feature importance for cluster 2.

**Figure 6:**
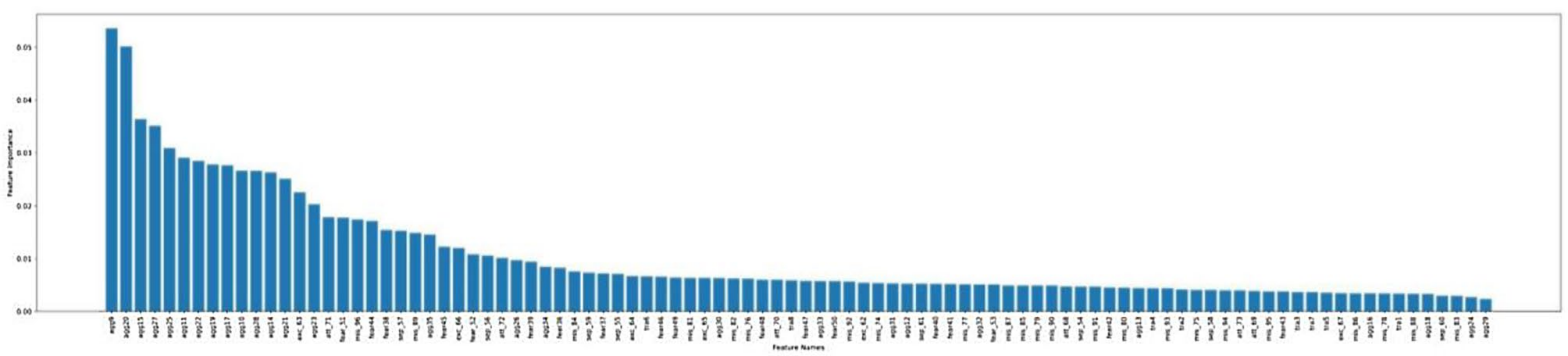
Feature importance for cluster 3.

**Figure 7:**
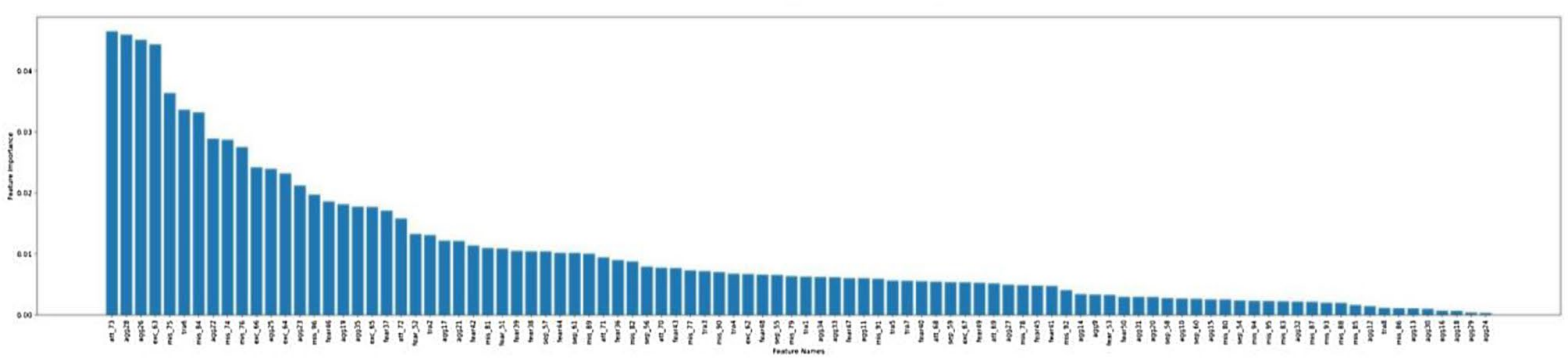
Feature importance for cluster 4.

Figures 3, 4, 5, 6, 7 demonstrate the feature importance in each cluster.

### Description of dog personality types

Five distinct groupings or categories of dogs emerged from the K-Means clustering analysis of behavioural data, corresponding to five different personality types. Dogs in Cluster 0 were characterized by relatively high levels of excitability, attachment/attention-seeking behavior and separation-related anxiety, and reduced fear compared with those in the other clusters. This personality type was labeled, “Excitable/Hyperattached.” Dogs in Cluster 1, in contrast, displayed relatively high levels of fear of both social (unfamiliar people, other dogs, etc.) and nonsocial (novel or unexpected situations or events) stimuli, and were labeled “Anxious/Fearful.” Cluster 2 dogs were labeled, “Aloof/Predatory” in recognition of their low levels of attachment/attention-seeking, and higher levels of predatory behavior and aggression toward other dogs, while Cluster 3 dogs were labeled “Reactive/Assertive” due to their heightened aggressive behavior across multiple domains, including aggression toward household members. Finally, dogs in Cluster 4 displayed consistently low levels of aggression, fear, excitability, and predatory behavior, and were labeled “Calm/Agreeable.” The ability to learn new tricks or tasks quickly was also typical of dogs in this cluster. These relationships are shown in Tables 3, 4, 5, 6 and 7 together with the feature importance of the items in each cluster.

**Table 3:** Cluster 0: Excitable/Hyperattached.

C-BARQ item	Item description	Feature importance	Cluster 0 means	Mean of other clusters
mis90	Hyperactive, restless, trouble settling down	0.042943	1.30 +	0.58
sep61	Loss of appetite when left alone	0.034686	0.42 +	0.32
att72	Jealous when owner gives attention to other people	0.031540	1.41 +	0.68
exc66	Excitable when taken on car trips	0.030422	2.47 +	1.6
mis91	Playful, puppyish, boisterous	0.027968	2.68 +	1.69
exc64	Excitable when doorbell rings	0.026235	2.68 +	2.01
mis89	Defecates when left alone	0.025326	0.28 +	0.21
att71	Nudges, paws owner for attention	0.024457	2.66 +	1.88
exc62	Excitable when owner comes home after absence	0.024042	2.93 +	2.1
mis84	Nervous or frightened on stairs	0.023624	0.26−	0.3
sep56	Restless, agitated when left alone	0.022145	1.12 +	0.63
exc65	Excitable before taken for walks	0.022010	2.72 +	1.84
agg35	Aggression when approached while playing with/chewing a favorite toy, bone, object, etc., by another (familiar) household dog	0.019635	0.68−	0.7
fear39	Fearful when unfamiliar persons visit your home	0.019525	0.25−	0.65
att70	Sits close to or in contact with owner	0.019257	3.17 +	2.54
fear38	Fearful in response to sudden or loud noises (e.g., vacuum cleaner, car backfire, road drills, objects being dropped, etc.)	0.016524	1.14−	1.18
fear36	Fearful when approached directly by an unfamiliar adult while away from your home	0.016307	0.32−	0.79
mis81	"Humps" objects, people, etc	0.015368	0.49 +	0.27
exc63	Excitable when playing with family members	0.014788	2.65 +	1.88
sep57	Whining when left alone	0.014559	1.54 +	0.84

+  = Cluster mean higher than combined mean of other clusters.

− = Cluster mean lower than combined mean of other clusters.

**Table 4:** Cluster 1: fearful/anxious.

C-BARQ item	Item description	Feature Importance	Cluster 1 mean	Mean of other clusters
agg35	Aggression when approached while playing with/chewing a favorite toy, bone, object, etc., by another (familiar) household dog	0.086189	0.60−	0.71
fear39	Fearful when unfamiliar persons visit your home	0.072337	1.82 +	0.39
fear38	Fearful in response to sudden or loud noises (e.g. vacuum cleaner, car backfire, road drills, objects being dropped, etc	0.059492	2.30 +	1.02
fear36	Fearful when approached directly by an unfamiliar **adult** while away from your home	0.049489	2.17 +	0.48
fear46	Fearful when approached directly by an unfamiliar dog of a smaller size	0.033463	0.85 +	0.44
fear37	Fearful when approached directly by an unfamiliar **child** while away from your home	0.030953	1.98 +	0.46
fear42	Fearful in response to strange or unfamiliar objects on or near the sidewalk (e.g. plastic trash bags, leaves, litter, flags flapping, etc	0.029978	1.23 +	0.43
fear40	Fearful when an unfamiliar person tries to touch or pet the dog	0.017972	2.23 +	0.48
fear47	Fearful when first exposed to unfamiliar situations (e.g. first car trip, first time in elevator, first visit to veterinarian, etc	0.017718	2.01 +	0.76
agg28	Aggression toward unfamiliar persons visiting your home	0.017328	0.82 +	0.47
fear41	Fearful in heavy traffic	0.017177	1.25 +	0.41
agg22	Aggression when joggers, cyclists, rollerbladers or skateboarders pass your home while your dog is outside or in the yard	0.015789	0.85 +	0.75
agg26	Aggression toward unfamiliar dogs visiting your home	0.013937	0.72−	0.83
fear43	Fearful when examined/treated by a veterinarian	0.013842	2.07 +	0.86
att73	“Jealous” when owner gives attention to another dog or animal	0.013215	1.43 +	1.41
agg23	Aggressive when approached directly by an unfamiliar **male** dog while being walked/exercised on a leash	0.013151	0.75−	0.89
mis75	Chases or would chase birds given the opportunity	0.012632	1.73−	1.98
agg25	Aggressive when stared at directly by a member of the household	0.010707	0.06−	0.05
mis90	Hyperactive, restless, has trouble settling down	0.010570	0.70−	0.74
exc66	Excitable just before taken on car trip	0.010514	1.81 +	1.79

+  = Cluster mean higher than combined mean of other clusters.

− = Cluster mean lower than combined mean of other clusters.

**Table 5:** Cluster 2: aloof/predatory.

C-BARQ item	Item description	Feature importance	Cluster 2 mean	Mean of other clusters
agg28	Aggression toward unfamiliar persons visiting your home	0.046446	0.44−	0.53
agg26	Aggression toward unfamiliar dogs visiting your home	0.040469	1.33 +	0.69
att73	Becomes agitated (whines, jumps up, tries to intervene) when you (or others) show affection for another dog or animal	0.037789	1.07−	1.5
agg22	Aggression when joggers, cyclists, rollerbladers or skateboarders pass your home while your dog is outside or in the yard	0.029619	0.89 +	0.73
att68	Displays strong attachment toward one particular member of household	0.029430	2.31−	2.77
agg25	Aggression when stared at directly by a member of the household	0.028940	0.03−	0.06
mis75	Chases or would chase birds given the opportunity	0.027460	2.46 +	1.82
agg23	Aggression when approached directly by an unfamiliar **male** dog while being walked/exercised on a leash	0.026174	1.42 +	0.73
sep61	Loss of appetite when left alone	0.019876	0.22−	0.38
att69	Tends to follow you (or other members of household) about the house, from room to room	0.019444	2.13−	2.84
mis89	Urinates when left alone at night, or during the daytime	0.019294	0.18−	0.23
sep56	Shows restlessness/agitation/pacing when left alone	0.018138	0.38−	0.83
mis74	Chases or would chase cats given the opportunity	0.017561	2.95 +	1.97
att72	Becomes agitated (whines, jumps up, tries to intervene) when you (or others) show affection for another person	0.016981	0.47−	0.93
att70	Tends to sit close to, or in contact with, you (or others) when you are sitting down	0.016138	2.22−	2.79
mis90	Hyperactive, restless, has trouble settling down	0.015776	0.41−	0.82
fear39	Fearful when unfamiliar persons visit your home	0.015195	0.28−	0.64
agg35	Aggression when approached while playing with/chewing a favorite toy, bone, object, etc., by another (familiar) household dog	0.014958	0.95 +	0.63
att71	Tends to nudge, nuzzle or paw you (or others) for attention when you are sitting down	0.014278	1.67−	2.15
exc62	Excitable when you or other members of the household come home after a brief absence	0.013771	1.95−	2.36

+  = Cluster mean higher than combined mean of other clusters.

− = Cluster mean lower than combined mean of other clusters.

**Table 6:** Cluster 3: reactive/assertive.

C-BARQ item	Item description	Feature importance	Cluster 3 mean	Mean of other clusters
agg9	Aggression when verbally corrected or reprimanded (scolded, shouted at, etc.) by you or a household member	0.053578	0.54 +	0.09
agg20	Aggression when strangers walk past your home while your dog is outside or in the yard	0.050147	2.11 +	0.75
agg15	Aggression when an unfamiliar person approaches you or another member of your family at home	0.036353	1.81 +	0.39
agg27	Aggression toward cats, squirrels or other small animals entering your yard	0.035104	2.29 +	1.03
agg25	Aggression when stared at directly by a member of the household	0.030881	0.24 +	0.03
agg11	Aggression when approached directly by an unfamiliar **child** while being walked/exercised on a leash	0.029025	1.42 +	0.20
agg22	Aggression when joggers, cyclists, rollerbladers or skateboarders pass your home while your dog is outside or in the yard	0.028445	2.04 +	0.60
agg19	Aggression when his/her food is taken away by a household member	0.027754	0.46 +	0.08
agg17	Aggression when approached directly by a household member while s/he (the dog) is eating	0.027611	0.38 +	0.07
agg10	Aggression when approached directly by an unfamiliar adult while being walked/exercised on a leash	0.026578	1.64 +	0.26
agg28	Aggression toward unfamiliar persons visiting your home	0.026567	1.74 +	0.36
agg14	Aggression when bathed or groomed by a household member	0.026246	0.44 +	0.08
agg21	Aggression when an unfamiliar person tries to touch or pet the dog	0.025054	1.64 +	0.21
exc63	Excitable when playing with you or other members of your household	0.022577	2.60 +	1.97
agg23	Aggression when approached directly by an unfamiliar **male** dog while being walked/exercised on a leash	0.020306	2.39 +	0.69
att71	Tends to nudge, nuzzle or paw you (or others) for attention when you are sitting down	0.017842	2.68 +	1.97
fear51	Fearful when having his/her feet toweled by a member of the household	0.017742	0.78 +	0.26
mis96	Chases/follows shadows, light spots, etc	0.017425	0.72 +	0.33
fear44	Fearful during thunderstorms, firework displays, or similar events	0.017130	1.56 +	1.00
fear38	Fearful in response to sudden or loud noises (e.g. vacuum cleaner, car backfire, road drills, objects being dropped, etc.)	0.015425	2.02 +	1.06

+  = Cluster mean higher than combined mean of other clusters.

− = Cluster mean lower than combined mean of other clusters.

**Table 7:** Cluster 4: calm/agreeable.

C−BARQ item	Item description	Feature importance	Cluster 4 mean	Mean of other clusters
att73	Becomes agitated (whines, jumps up, tries to intervene) when you show affection for another dog or animal	0.046477	0.26−	1.72
agg28	Aggression toward unfamiliar persons visiting your home	0.045940	0.17−	0.69
agg26	Aggression toward unfamiliar dogs visiting your home	0.045085	0.27−	1.11
exc63	Excitable when playing with you or other members of your household	0.044342	1.68−	2.23
mis75	Chases or would chase birds given the opportunity	0.036373	1.15−	2.38
tra6	Quick to learn new tricks or tasks	0.033636	3.10 +	2.74
mis84	Nervous or frightened on stairs	0.033216	0.16−	0.35
agg22	Aggression when joggers, cyclists, rollerbladers or skateboarders pass your home while your dog is outside or in the yard	0.028883	0.30−	1.01
mis74	Chases or would chase cats given the opportunity	0.028717	1.34−	2.62
mis76	Chases or would chase squirrels, rabbits and other small animals given the opportunity	0.027501	1.83−	3.06
exc66	Excitable just before being taken on a car trip	0.024193	1.20−	2.11
agg25	Aggression when stared at directly by a member of the household	0.023929	0.01−	0.08
exc64	Excitable when doorbell rings	023,186	1.39−	2.56
agg23	Aggression when approached directly by an unfamiliar **male** dog while being walked/exercised on a leash	0.021218	0.27−	1.2
mis96	Chases/follows shadows, light spots, etc	0.019714	0.22−	0.46
fear46	Fearful when approached directly by an unfamiliar dog of a smaller size	0.018595	0.16−	0.66
agg19	Aggression when his/her food is taken away by a household member	0.018146	0.04−	0.17
agg35	Aggression when approached while playing with/chewing a favorite toy, bone, object, etc., by another (familiar) household dog	0.017738	0.33−	0.89
exc65	Excitable just before being taken for a walk	0.017680	1.42−	2.36
fear37	Fearful when approached directly by an unfamiliar **child** while away from your home	0.017085	0.26−	0.84

+  = Cluster mean higher than combined mean of other clusters.

− = Cluster mean lower than combined mean of other clusters.

### Evaluating the performance of machine learning classifiers

**Figure 8:**
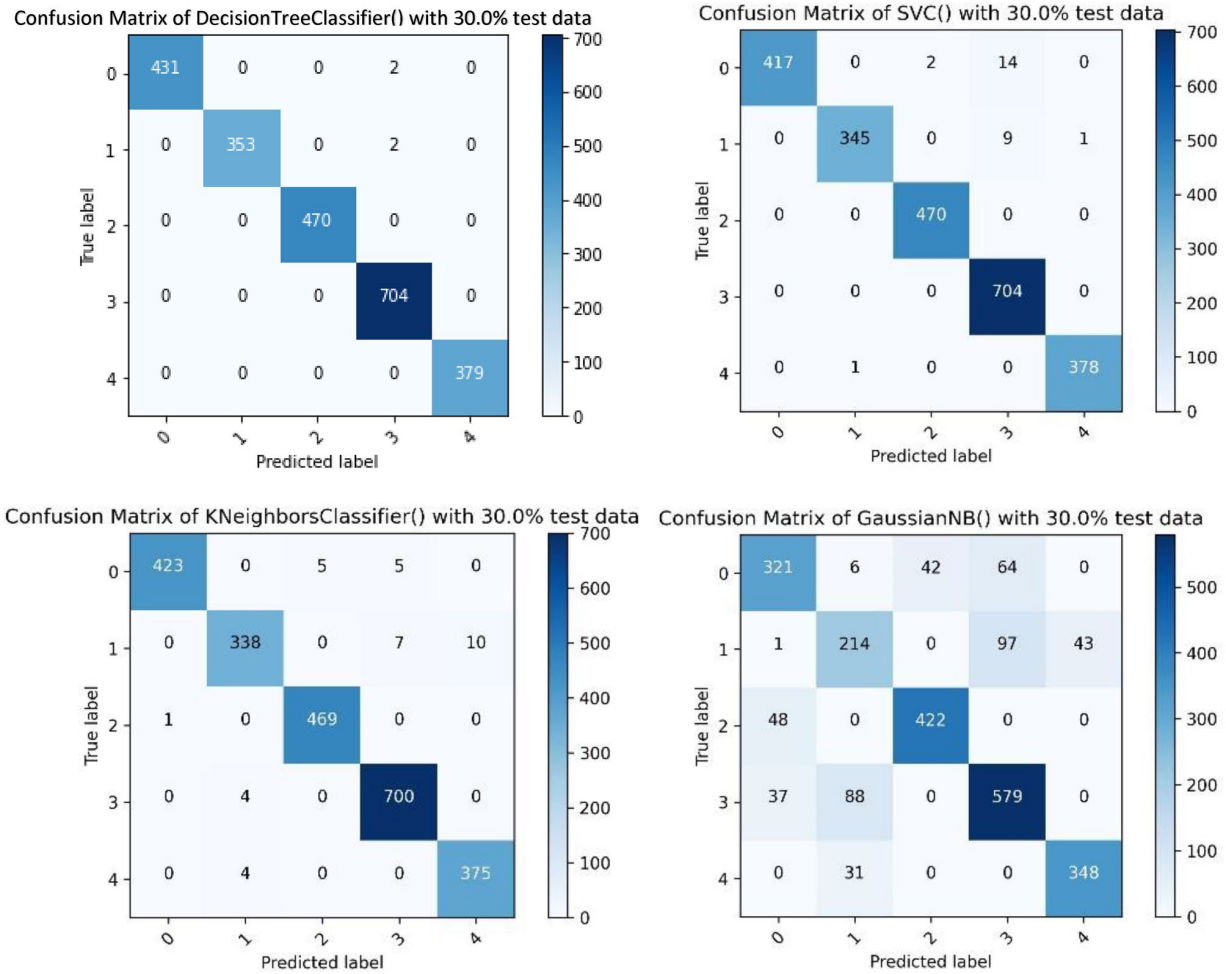
Confusion matrices for Decision Tree, Naïve Bayes, KNN and SVM models.

The four confusion matrices presented in Fig. 8 visualise the performance of the trained models when the dataset was divided into 70% for training and 30% for testing. The confusion matrix for these models highlights the multi-class classification of this work, where the target variable has five values in the range of 0 to 4 representing different personality types. The columns represent the predicted values of the target variable and the rows represent the actual values of the target variable.

In a confusion matrix for a multiclass classification problem, the terms True Positive ($TP$), True Negative ($TN$), False Positive ($FP$), and False Negative ($FN$) are defined as follows:

True Positive ($TP$): The number of instances of class $i$ that were correctly predicted as class $i$.

True Negative ($TN$): The number of instances not belonging to class $i$ that were correctly predicted as not belonging to class $i$.

False Positive ($FP$): The number of instances not belonging to class $i$ that were incorrectly predicted as belonging to class $i$.

False Negative ($FN$): The number of instances of class $i$ that were incorrectly predicted as not belonging to class $i$.

According to Fig. 8, the Decision Tree model has the highest TP value for the class ‘0’, ‘1’ and ‘4’. Both decision Tree and Support Vector Machine have the highest number of correct predictions for class ‘2’. Support Vector Machine has the highest TP value for class ‘3’.

Table 8 shows the calculated precision, recall and F1 score for each class per model used. Following this step, the accuracy percentage was calculated for each model and the results are presented in Table 9. In the realm of machine learning, Precision is the ratio of true positives to the sum of true positives and false positives. It measures the accuracy of positive predictions. Precision can be calculated using this formula:

**Table 8:** Precision, Recall, and F1 score for each class and model.

Classifier	Class	Precision	Recall	F1 score
SVM	0	1	0.96	0.98
	1	1	0.97	0.98
	2	1	1	1
	3	0.97	1	0.98
	4	1	1	1
KNN	0	1	0.98	0.99
	1	0.98	0.95	0.96
	2	0.99	1	0.99
	3	0.98	0.99	0.99
	4	0.97	0.99	0.98
Decision tree	0	1	1	1
	1	1	0.99	1
	2	1	1	0.99
	3	0.99	1	1
	4	1	1	1
Naïve bayes	0	0.79	0.74	0.76
	1	0.63	0.6	0.62
	2	0.91	0.9	0.9
	3	0.78	0.82	0.8
	4	0.89	0.92	0.9

**Table 9:** Accuracy for each model.

Classifier	Accuracy percentage (%)
SVM	98
KNN	98
Decision tree	99
Naïve bayes	79

8 $$Precision= \frac{TP}{TP+FP}$$

In addition, Recall is the ratio of true positives to the sum of true positives and false negatives. It quantifies the ability of the classifier to capture all positive instances. Recall can be calculated using the following formula:

9 $$Recall= \frac{TP}{TP+FN}$$

The F1 score is a metric that combines precision and recall into a single value, providing a balanced assessment of a classification model’s performance. The formula for calculating the F1 score is as follows:

10 $$F1=2 \times \frac{Precision \times Recall}{Precision+Recall}$$

Finally, the accuracy of a model, serves as a fundamental metric gauging its overall performance. It is computed by discerning the ratio of correct predictions, represented by the sum of true positives ($TP$) and true negatives ($TN$), to the aggregate number of predictions. Mathematically, the accuracy is expressed as:

11 $$Accuracy= \frac{TP+TN}{TP+TN+FP+FN}$$

To obtain an overall accuracy for a multiclass problem, you can average the accuracies across all classes.

The Precision, Recall, F1 score, and Accuracy have been calculated using the Scikit-learn Python library that provides functions to calculate these evaluation metrics automatically. 

The Decision Tree model demonstrated the best performance amongst the four models investigated. The SVM and KNN have the same results, while the Naïve Bayes model shows the weakest performance. For a more in-depth evaluation of the model, the five-fold cross validation was performed in which models were run for 5 times on different random selections of data to evaluate their performance. For the cross-validation experiments, accuracy was reported as the evaluation metric, so the accuracy percentage could be compared with the presented results in Table 9. Table 10 shows the accuracy scores calculated for each fold and the mean classification accuracy as the final accuracy score for each model.

**Table 10:** fivefold cross validation results for each model.

Classifier	Fold number	Accuracy scores calculated for each fold	Mean accuracy score
SVM	1 2 3 4 5	0.98507463 0.98290598 0.98504274 0.98290598 0.99145299	0.98
KNN	1 2 3 4 5	0.97654584 0.97649573 0.98076923 0.98290598 0.97435897	0.97
Decision tree	1 2 3 4 5	0.98933902 0.9957265 0.98076923 0.99786325 0.99786325	0.99
Naïve bayes	1 2 3 4 5	0.76332623 0.76282051 0.78205128 0.77350427 0.79487179	0.77

According to Table 10, the overall performance of the SVM and decision Tree models remained the same. The performance of KNN model dropped by 1% and the performance of Naïve Bayes model dropped by 2%. Additionally, Table 11 shows the comparison between five-Fold cross validation results and the accuracy scores from Table 9.

**Table 11:** Accuracy for each model.

Classifier	Accuracy percentage (%)	Mean accuracy score after five-fold cross validation (%)
SVM	98	98
KNN	98	97
Decision tree	99	99
Naïve bayes	79	77

The mean accuracy score was considered as the final accuracy score after performing five-fold cross validation. As a result, Table 11 shows that the Decision Tree model has the best performance with an accuracy of 99%. SVM is the second model with an accuracy of 98%. KNN with accuracy of 97% and Naïve Bayes with 77%, respectively, are the third and fourth performing models. Furthermore, it can be confidently claimed that the results are not biased as the performance of the models has been evaluated in 5 different iterations using different parts of the dataset with the same size at each step of the validation process.

## Discussion

Artificial Intelligence and Machine Learning (ML) techniques have been used effectively for classifying and predicting human personality types, but similar approaches have not yet been applied to analysing and predicting canine personality. Using K-Means algorithm and Feature Importance analysis of behavioral survey data we were able to identify five main personality groupings in dogs which we labelled: “Excitable/Hyperattached” (cluster 0), “Anxious/Fearful” (cluster 1), “Aloof/Predatory” (cluster 2), “Reactive/Assertive” (cluster 3), and “Calm/Agreeable” (cluster 4) based on the behavioral variables that contributed most to each trait. Descriptions of each of the personality types were generated based on calculating the mean value for the top 20 most important features extracted for each cluster and comparing them with the mean value of the same attributes in the other four clusters combined. An important difference between these ML techniques and the methods used to develop more traditional personality assessments, such as the Big Five Inventory, is that, while the former aggregate or cluster individual dogs according to similarities in their reported behaviour (C-BARQ scores), the latter typically involves grouping questionnaire items with correlated scores to create personality ‘traits’ that can be used to describe or profile individuals.

Taken at face value, these personality clusters appear to be biologically meaningful, in the sense that they describe broad domains of canine temperament that would be recognizable to a majority of dog owners and handlers. Clusters 1, 3 and 4 resemble previous subgroups of dogs identified from C-BARQ data using Latent Class Analysis^37^, as well as canine personality factors found in previous studies using both questionnaire and behavioral testing methods^14,52^. The “excitability” component of cluster 0 also overlaps with the trait labelled “extraversion” by Ley et al.^17^ and “behavioural regulation” by Wright et al.^53^, although these factors do not include behaviors related to attachment or attention-seeking, as in our findings^14^. Clusters 1 and 4 in the present analysis are also comparable to the human “Big Five” personality factors “neuroticism” and “agreeableness,” respectively, but there are fewer obvious parallels between our “excitable/hyperattached”, “aloof/predatory” and “reactive/assertive” canine personalities and any of the Big Five personality types, suggesting that these clusters may be specific to dogs. In future research, it would be valuable to determine how the membership of these distinct canine personality clusters is predicted or influenced by demographic and background variables such as age, sex, body size, neuter status, breed, previous history, and characteristics of the environment including the personality and experience of the owner/handler^54^. It would also be of considerable interest to identify any genetic associations with cluster membership.

As with MBTI personality traits in humans which can be used to guide individuals toward appropriate careers based on their personality characteristics, the methods presented in this paper may provide a framework for evaluating the suitability of individual dogs for specific working and/or social roles. For example, previous studies of different types of working dogs have identified shyness/anxiety as one of the most common reasons for poor working performance^52,55^. This would suggest that dogs classified as “anxious/fearful” (cluster 1) by our methods would be less likely to be successful in the majority of working careers. Conversely, it is widely recognized that the ‘ideal’ assistance dog (ie., guide and service dog) tends to have a very different personality from the ideal odor detection dog^56,57^, and it would be valuable to know whether high (or low) performing dogs in either of these working categories could be predicted based on one or more of the other four personality clusters identified here. Currently, many working dog organizations use the C-BARQ to collect behavioral information about their dogs during their first year of life^15,22^, so future analyses of this type are certainly feasible, and could provide an opportunity to screen younger dogs for eligibility as future breeding animals or for working roles. The proposed methods could also be used to explore personality matching between companion dogs and their owners and how this might contribute to the quality and durability of their relationships. The results of such studies could potentially generate insights regarding why dog-human partnerships succeed or fail, thereby reducing future rates of shelter relinquishment and euthanasia, and may also help to guide animal shelter and rescue groups towards more successful and mutually rewarding dog adoptions. Similarly, in the fields of dog training and behavior modification, the ML methods described here could potentially be used to direct and enhance remedial approaches to canine behavior problems that take account of each animal’s underlying personality type.

Four machine learning models were implemented in this research to predict the personality types of dogs. The Decision Tree model showed the best performance amongst the four models investigated. The SVM and KNN had the same results, while the Naïve Bayes model showed the weakest performance. After performing the five-fold cross validation, the overall performance of the SVM and Decision Tree models remained the same. The performance of KNN model dropped by 1% and the performance of Naïve Bayes model dropped by 2%.

The mean accuracy score was considered as the final accuracy score after performing five-fold cross validation. Again, the Decision Tree model showed the best performance with an accuracy of 99% versus 98% for the SVM, 97% for the KNN, and 77% for the Naïve Bayes. Furthermore, it can be confidently claimed that the results are not biased as the performance of the models has been evaluated in 5 different iterations using different parts of the dataset with the same size at each step of the validation process.

Although K-means algorithm is one of the most popular and successful unsupervised learning algorithms for clustering in different fields, it may have some limitations that must be considered when it is used on real-world datasets. One of the limitations of this algorithm is that the number of clusters should be specified a priori, which can be a challenging task, especially when the dataset does not have a clear structure. Choosing an inappropriate number of clusters can lead to suboptimal clustering results. To tackle this challenge, the Elbow method was used in this research which demonstrated that the optimum value for the number of clusters is 5. However, the algorithm was also tested with initial values of 4 and 6 for the number of clusters and the results showed that 5 is the best value for the number of clusters as data points are clearly separated and allocated to different clusters. In addition, this unsupervised algorithm is sensitive to outliers, as they can distort the centroid of a cluster and lead to suboptimal clustering results. The presence of noise or outliers in the dataset can also impact the performance of the supervised learning algorithms. For this reason, the first stage of this research involved cleaning the original C-BARQ dataset to ensure that the performance of both unsupervised and supervised models used would not be impacted by outliers and noisy data.

Regarding the performance of the classification models used in this research, as the results show, the Naïve Bayes model has the worst accuracy for prediction compared to other models. The reason could be that this probabilistic model assumes features are completely independent and have a Gaussian distribution. This assumption may not always be valid, particularly when dealing with large-scale datasets with highly associated properties. Thus, the model may fail to recognise the underlying patterns in the data, resulting in poor classification performance and label prediction. The fact that KNN, SVM, and Decision Tree models outperform the Gaussian Naive Bayes model in this research may be attributed to the features being strongly correlated and having a nonlinear connection with the output. KNN, SVM, and Decision Tree models are more resistant to such circumstances and can capture nonlinear correlations between features and output.

While the current findings demonstrate the remarkable accuracy with which ML can assign dogs to specific personality categories based on their reported behavioral responses to common domestic situations and stimuli, further studies will be needed to determine how this information can best be applied in practice. This will likely depend on the intrinsic limitations of the data used to train the models. Although the C-BARQ is widely used and has demonstrated reliability and validity as a canine behavioral assessment tool^15,16,22–36^, the data it generates come from dog owners/handlers and are therefore inevitably susceptible to subjective biases and variation in reporting accuracy. Additionally, the survey items only address some aspects of behavior and may ignore others that are important to a comprehensive understanding of canine personality^16,55^. Future studies should consider these limitations when applying these methods in real world contexts and integrate them with, and validate them against, other more objective measures including direct behavioral observation and testing, output from motion/activity and biometric sensors, and assays of physiological indicators.

## Conclusion

Artificial Intelligence and Machine Learning techniques have been widely and successfully used for classifying and predicting human personality types based on different personality profiling tools such as the Myers Briggs Type Indicator (MBTI) and the Big Five Inventory (BFI). Here we describe the use of similar approaches to the analysis and prediction of canine personality using behavioural data derived from the C-BARQ.

Using an unsupervised learning approach and K-Means algorithm, five distinct clusters of dogs were identified, corresponding to five different personality types. These traits were labelled: “Excitable/Hyperattached”, “Anxious/Fearful”, “Aloof/Predatory”, “Reactive/Assertive”, and “Calm/Agreeable” based on the different C-BARQ behavioral variables’ relative contributions (Feature Importance) to the ML models. The performance of the models was evaluated using five-fold cross validation method and the results showed that the Decision Tree model had the best performance with an accuracy of 99%.

The methods developed in this study have the potential to provide a useful tool in the selection and training of both working and non-working dogs, though additional research is needed to assess their validity in specific canine populations.

## Data Availability

The data that support the findings of this study are available from the third author upon reasonable request.
